# Meperidine pharmacokinetics and effects on physiologic parameters and thermal threshold following intravenous administration of three doses to horses

**DOI:** 10.1186/s12917-020-02564-4

**Published:** 2020-10-01

**Authors:** Briana D. Hamamoto-Hardman, Eugene P. Steffey, Daniel S. McKemie, Philip H. Kass, Heather K. Knych

**Affiliations:** 1 K.L. Maddy Equine Analytical Pharmacology Laboratory, School of Veterinary Medicine, University of California-Davis, CA 95616 Davis, USA; 2 Department of Veterinary Surgery and Radiology, School of Veterinary Medicine, University of California, Davis, USA; 3 Department of Population Health and Reproduction, School of Veterinary Medicine, University of California, Davis, USA; 4 Department of Veterinary Molecular Biosciences, School of Veterinary Medicine, University of California, Davis, USA

**Keywords:** Horse, Meperidine, Opioid, Pharmacokinetics, Pharmacodynamics, Thermal threshold

## Abstract

**Background:**

Meperidine is a synthetic opioid that belongs to the phenylpiperidine class and is a weak mu receptor agonist. In horses there are a limited number of published studies describing the analgesic effects of systemically administered meperidine in horses. The objective of this study was to describe the pharmacokinetics, behavioral and physiologic effects and effect on thermal threshold of three doses of intravenously administered meperidine to horses. Eight University owned horses (four mares and four geldings, aged 3–8 years were studied using a randomized balanced 4-way cross-over design. Horses received a single intravenous dose of saline, 0.25, 0.5 and 1.0 mg/kg meperidine. Blood was collected before administration and at various time points until 96 hours post administration. Plasma and urine samples were analyzed for meperidine and normeperidine by liquid chromatography-mass spectrometry and plasma pharmacokinetics determined. Behavioral and physiologic data (continuous heart rate, step counts, packed cell volume, total plasma protein and gastrointestinal sounds) were collected at baseline through 6 hours post administration. The effect of meperidine administration on thermal nociception was determined and thermal excursion calculated.

**Results:**

Meperidine was rapidly converted to the metabolite normeperidine. The volume of distribution at steady state and systemic clearance (mean ± SD) ranged from 0.829 ± 0.138–1.58 ± 0.280 L/kg and 18.0 ± 1.4–22.8 ± 3.60 mL/min/kg, respectively for 0.5–1.0 mg/kg doses. Adverse effects included increased dose-dependent central nervous excitation, heart rate and cutaneous reactions. Significant effects on thermal nociception were short lived (up to 45 minutes at 0.5 mg/kg and 15 minutes at 1.0 mg/kg).

**Conclusions:**

Results of the current study do not support routine clinical use of IV meperidine at a dose of 1 mg/kg to horses. Administration of 0.5 mg/kg may provide short-term analgesia, however, the associated inconsistent and/or short-term adverse effects suggest that its use as a sole agent at this dose, at best, must be cautiously considered.

## Background

With the exception of drugs used to treat pain associated with inflammation, in equine medicine, there are a limited number of analgesic drugs that have been fully characterized. Opioids are potent analgesics in many species, making them an attractive choice for pain management in horses. However, IV administration of opioids to horses is often times associated with dose- dependent moderate to excessive central nervous system stimulation that can manifest as agitation and increased locomotion [1, 2].

Meperidine is a synthetic opioid that belongs to the phenylpiperidine class and is a weak mu receptor agonist [3]. Reports in humans suggest that meperidine provides comparable analgesia to morphine, albeit with a shorter duration of analgesic effect [4]. In horses there are a limited number of published studies describing the analgesic effects of systemically administered meperidine [5–7]. In one such study, investigators reported a short duration of analgesia, compared to saline, following intramuscular injection of meperidine (1 mg/kg) in a model of foot pain [5]. In a second study, although the duration of analgesic effect was shorter than other commonly used analgesics, meperidine was found to be effective in the treatment of visceral pain in balloon- induced model of colic [6]. In a limited survey study [7] noted a decrease in the minimum alveolar concentration of isoflurane necessary to prevent movement following application of a noxious stimuli when horses were administered meperidine (2.0 mg/kg, IV) during isoflurane anesthesia. Following caudal epidural administration, meperidine elicits long- lasting perineal analgesia [8, 9]. Although reports are limited, adverse effects range from minimal sedation, ataxia and cardiopulmonary effects following epidural administration [9] to head shaking, itching, ataxia and agitation following systemic administration [10].

The pharmacokinetics of meperidine in horses has been minimally studied [10, 11]. Waterman and Amin [10] described the pharmacokinetics of meperidine (1 mg/ kg IV) both prior to and immediately following recovery from general anesthesia. In that study, meperidine was characterized by a short elimination half-life with prior anesthetic administration impacting its pharmacokinetic properties. In rats, humans and non-human primates, one of the metabolites produced following meperidine administration is the active metabolite, normeperidine [12–14]. Although in humans, normeperidine has only half the analgesic potency of meperidine, it also reportedly has neuroexcitatory properties [15] with higher plasma normeperidine to meperidine metabolic ratios associated with more severe CNS effects, including seizures and death [16, 17]. This neuroexcitation does not appear to be mediated by u-receptors. To the best of the authors’ knowledge, the pharmacokinetics of normeperidine has not been reported previously in horses.

The potential analgesic effects of meperidine, coupled with limited reports of adverse effects and pharmacokinetics, supports further study of this compound in horses. To that end, the objective of the current study was to describe the pharmacokinetics and clinically important pharmacodynamic effects, including response to noxious stimulation, of IV meperidine in horses.

## Results

### Pharmacokinetics

The LC-MS/MS instrument responses for meperidine and normeperidine were linear and yielded correlation coefficients of 0.99 or better. The analyst to analyst precision and accuracy of the assay was determined by assaying quality control samples in replicates (*n* = 6). Accuracy was reported as percent nominal concentration, and precision was reported as percent relative standard deviation (Table 1).

**Table 1:** Accuracy and precision values for LC-MS/MS analysis of meperidine and normeperidine in plasma and urine

Drug	Matrix	Concentration (µg/mL)	Intra-day accuracy (% nominal concentration)	Intra-day precision (% relative SD)	Inter-day accuracy (% nominal concentration)	Inter-day precision (% relative SD)
Meperidine						
	Plasma	0.3	113	3.0	106	2.0
		40.0	100	1.0	103	1.0
		1000	88.0	2.0	89.0	3.0
	Urine	0.75	95.0	4.0	99.0	5.0
		50.0	100	3.0	103	10.0
		1000	100	4.0	104	5.0
Normeperidine						
	Plasma	0.3	103	4.0	99.0	5.0
		40.0	100	2.0	102	2.0
		1000	99.0	3.0	104	3.0
	Urine	0.75	103	3.0	101	5.0
		50.0	105	2.0	106	2.0
		1000	100	4.0	105	3.0

**Fig. 1:**
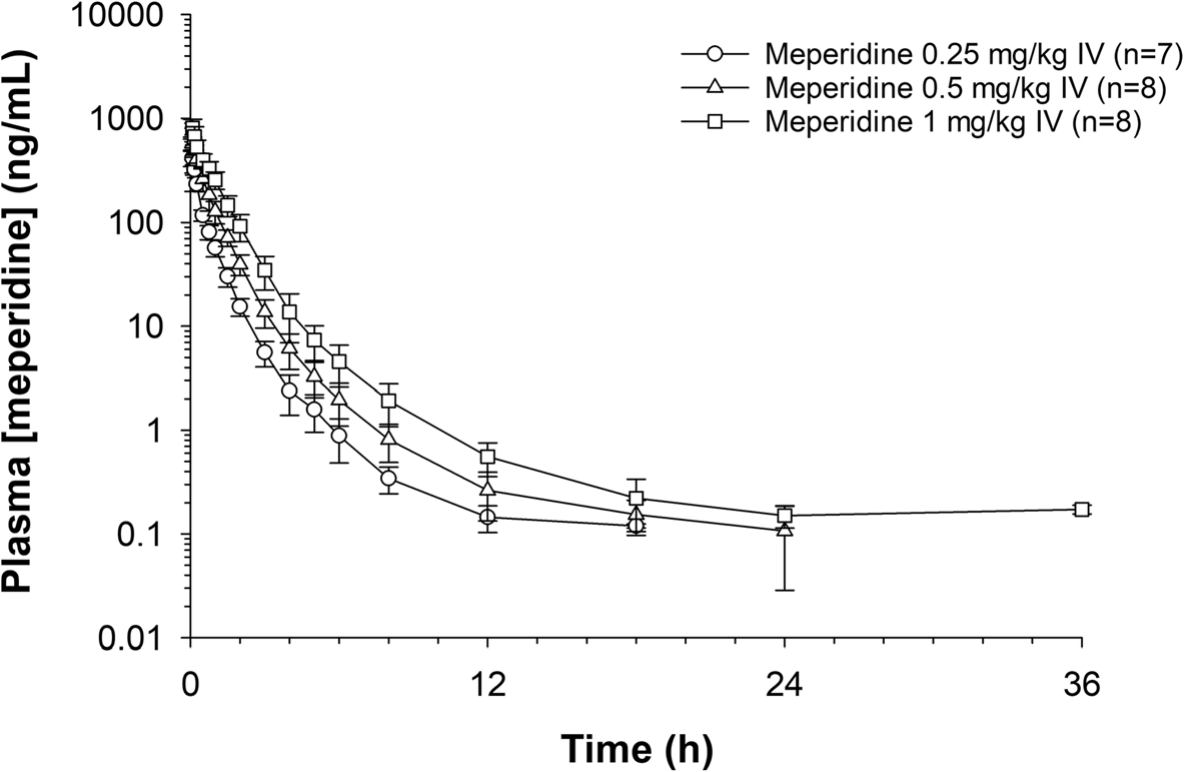
Average ± SD plasma concentrations of meperidine with respect to time after a single IV administration of meperidine HCl (0.25, 0.5 and 1.0 mg/kg) to eight horses

**Fig. 2:**
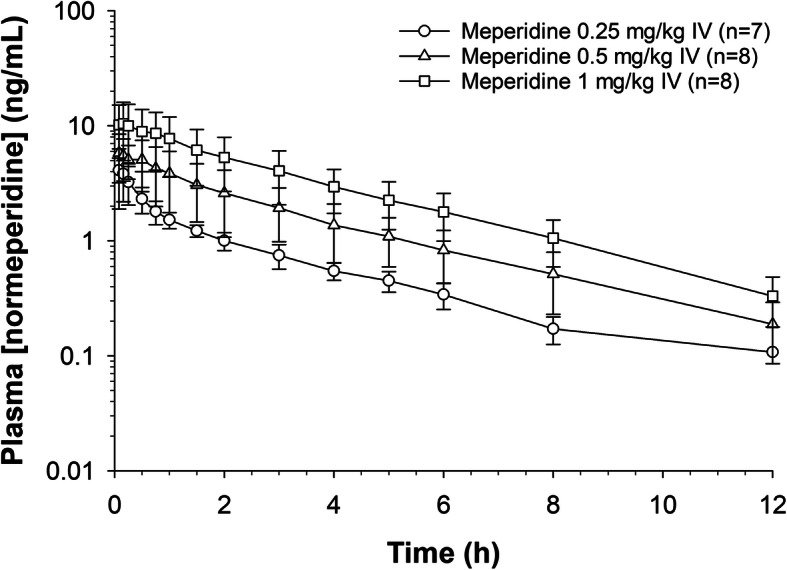
Average ± SD plasma concentrations of normeperidine with respect to time after IV administration of meperidine HCl (0.25, 0.5 and 1.0 mg/kg) to eight horses

Plasma concentration time curves for meperidine and normeperidine are depicted in Figs. 1 and 2. Concentrations of meperidine fell below the LOQ of the assay (0.10 ng/mL) by 18, 24 and 36 hours for the 0.25, 0.5 and 1 mg/kg dose groups, respectively. The average plasma normeperidine concentrations were below the LOQ (0.10 ng/mL) of the assay by 18 hours post meperidine administration in the 0.25 and 0.5 mg/kg dose groups and by 24 hours post administration in the 1 mg/kg dose group. Pharmacokinetic parameters for meperidine and normeperidine are listed in Tables 2 and 3, respectively. For meperidine, the Vd_ss_ was significantly different (*p* < .05) between all dose groups and systemic clearance was significantly (*p* < .05) higher in the 1 mg/kg dose group compared to the two lower dose groups. The elimination half-life was significantly different between the 0.25 and 0.5 mg/ kg dose groups only.

**Table 2:** Pharmacokinetic parameters (mean ± SD) for meperidine following a single IV administration of meperidine HCl (0.25, 0.5 and 1.0 mg/kg) to adult horses. All values reported were generated using non-compartmental analysis

Parameters	Dose Groups		
	0.25 mg/kg (*n* = 7)	0.5 mg/kg (*n* = 8)	1.0 mg/kg (*n* = 8)
C(0) ng/mL	540.9 ± 142.9^c^	691.9 ± 123.1^c^	987.7 ± 224.6^ab^
Lambda_z_(1/h)	0.358 ± 0.157^b^	0.245 ± 0.139^a^	0.207 ± 0.062
HL Lambda_z_ (h)	1.93 ± 1.39^c^	2.82 ± 2.10	3.35 ± 0.940^a^
Vdss (L/kg)	0.829 ± 0.138^bc^	1.08 ± 0.194a^c^	1.58 ± 0.280^ab^
CL (mL/min/kg)	18.0 ± 1.40^c^	19.3 ± 3.03^c^	22.8 ± 3.60^ab^
AUC_0 − inf_ (h*ng/mL)	232.7 ± 19.4^bc^	442.8 ± 73.1^ac^	748.3 ± 125.5^ab^

*C(0) *Concentration extrapolated to the origin, Lambda_z_, terminal slope; HL Lambda_z,_ terminal half-life, *Vdss *Volume of distribution at steady-state, *CL *Clearance, AUC_0 − inf_, Area under the plasma-concentration curve from time 0 to infinity

^a^significantly different (*p* < .05) from 0.25 mg/kg

^b^significantly different (*p* < .05) from 0.5 mg/kg

^c^significantly different (*p* < .05) from 1.0 mg/kg

**Table 3:** Pharmacokinetic parameters (mean ± SD) for normeperidine following a single IV administration of meperidine HCl (0.25, 0.5 and 1.0 mg/kg) to adult horses. All values reported were generated using non-compartmental analysis

Parameters	Dose Groups		
	0.25 mg/kg (*n* = 7)	0.5 mg/kg (*n* = 8)	1.0 mg/kg (*n* = 8)
C_max_ ng/mL	4.27 ± 2.07	6.23 ± 2.62	11.2 ± 5.64
T_max_ (h)	0.14 ± 0.06	0.22 ± 0.24	0.14 ± 0.06
Lambda_z_(1/h)	0.307 ± 0.010	0.272 ± 0.054	0.271 ± 0.054
HL Lambda_z_ (h)	2.39 ± 0.498	2.63 ± 0.449	2.63 ± 0.449
AUC_0 − inf_ (h*ng/mL)	7.35 ± 1.58	17.8 ± 9.26	35.6 ± 17.8

C_max_ maximum measured concentration; T_max,_ time of maximum concentration; Lambda_z_, terminal slope; HL Lambda_z,_ terminal half-life; AUC_0 − inf_, area under the plasma-concentration curve from time 0 to infinity

Urine concentrations of meperidine were no longer detectable by 24 hours in the 0.25 mg/kg group and by 48 hours in the 0.5 and 1.0 mg/kg groups (Table 4). Normeperidine concentrations fell below the limit of detection in all horses by 48 hours in the 0.25 mg/kg group and 72 hours in the 0.5 and 1.0 mg/kg dose groups (Table 4).

**Table 4:** Mean ± SD urine concentrations of meperidine and normeperidine following a single IV administration of meperidine HCl (0.25, 0.5 and 1.0 mg/kg) to adult horses

	Meperidine concentration (ng/mL)			Normeperidine concentration (ng/mL)		
	0.25 mg/kg(*n* = 7)	0.5 mg/kg(*n* = 8)	1.0 mg/kg(*n* = 8)	0.25 mg/kg(*n* = 7)	0.5 mg/kg(*n* = 8)	1.0 mg/kg(*n* = 8)
4 hours	135.2 ± 53.5	993.5 ± 1822.7	1142.6 ± 912.9	153.1 ± 120.4	956.8 ± 1646.6	1217.8 ± 1319.0
24 hours	ND	1.51 ± 0.82	4.42 ± 5.89	1.13 ± 0.17 (*n* = 3)	1.80 ± 0.60	4.70 ± 5.67
48 hours	ND	ND	ND	ND	0.37 ± 0 (*n* = 1)	0.48 ± 0.12 (*n* = 3)
72 hours	ND	ND	ND	ND	ND	ND
96 hours	ND	ND	ND	ND	ND	ND

*ND *Not detected

### Behavioral and Physiologic Responses

No adverse or abnormal behavioral responses were noted in the saline group throughout the study period. Behavioral reactions in the 0.25 mg/kg dose group included: muscle fasciculations in one horse (commencing within 15 minutes and observed intermittently for up to 36 hours post administration) following administration; sedation-like effects noted (glassy eyed appearance and quiet for extended periods of time) starting at 15 minutes and lasting up to 45 minutes in 4 horses and up to 4 hours in one horse; head shaking immediately upon administration in 3 horses; and intermittent circling (1 horse) from 5 minutes up to 1.5 hours post administration. Following administration of 0.5 mg/kg, reactions included: head shaking (1 horse) within 2.5 minutes of drug administration; sedation-like effects (3 horses) starting within 30 minutes and for up to 3 hours post administration; and excitation (circling) within 5 minutes in 2 horses. Horses in the 1 mg/kg dose group exhibited: pacing (3 horses) including trotting circles in the stall (1 horse); head shaking (6 horses); ataxia (3 horses); profuse sweating (2 horses); whole body tremors (4 horses); tail swishing (3 horses); and glazed (1 horse) and protruding eyes (4 horses).

**Fig. 3:**
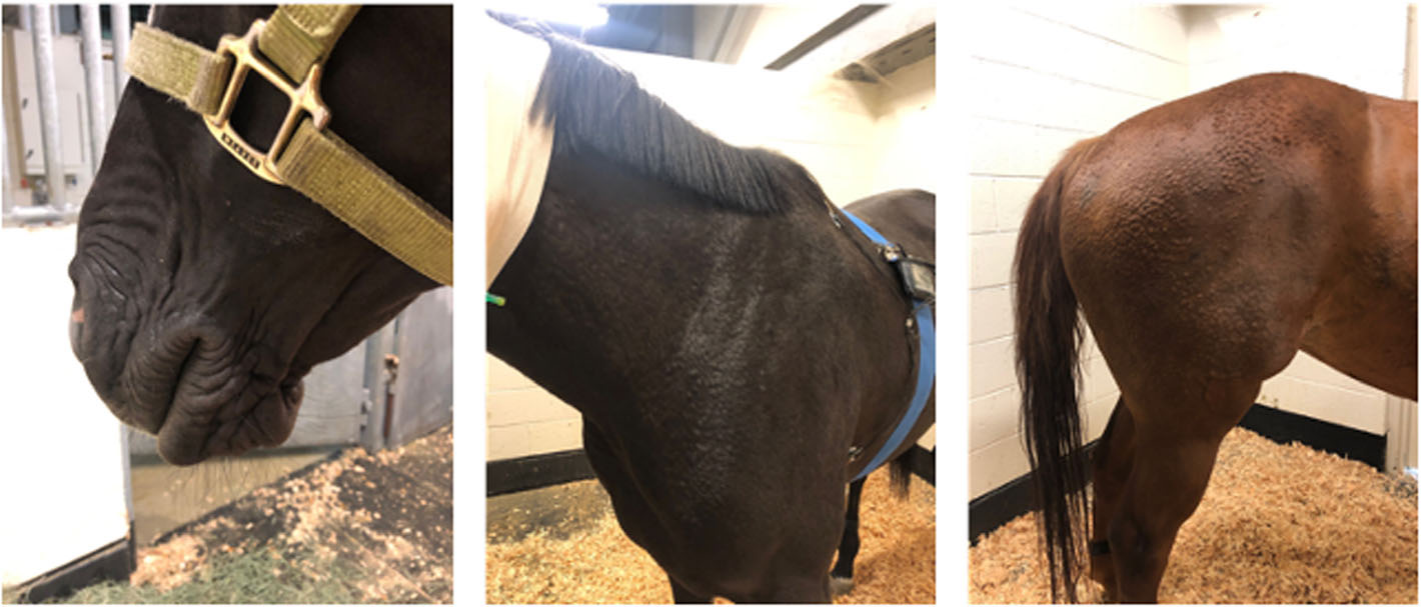
Characteristic cutaneous reactions observed following intravenous administration of meperidine to horses

Hives, covering the entire body (face, neck, chest and hindquarters) were noted in all dose groups but saline (0.25 mg/kg: 4 horses; 0.5 mg/kg: 8 horses; 1 mg/kg: 7 horses), with a notable increase in severity with increasing dose. In all dose groups, hives were noticeable within the first 2.5-5 minutes and started on the chest or neck and subsequently moved towards the face and/or hindquarters (Fig. 3). The persistence of hives varied between individual horses and between dose groups. In the 0.25 mg/kg group, hives were present for up to 15 minutes; following administration of 0.5 and 1 mg/kg, hives were noticeable for up to 1.5 hours. In 2 horses in the 1.0 mg/kg group, noticeable swelling in the throatlatch area necessitated removal of the elastikon bandage covering the catheter. Facial swelling (nose and eyes) and swelling between the hindlegs were also observed in a number of horses in the 1 mg/kg dose group. Several horses appeared very pruritic, rubbing themselves on the walls of the stall. All horses in the 1 mg/kg dose group began to sweat within 30 seconds to 1 minute of drug administration. At all doses for all horses, the hives eventually disappeared without any intervention (i.e. administration of anti-histamines).

**Fig. 4:**
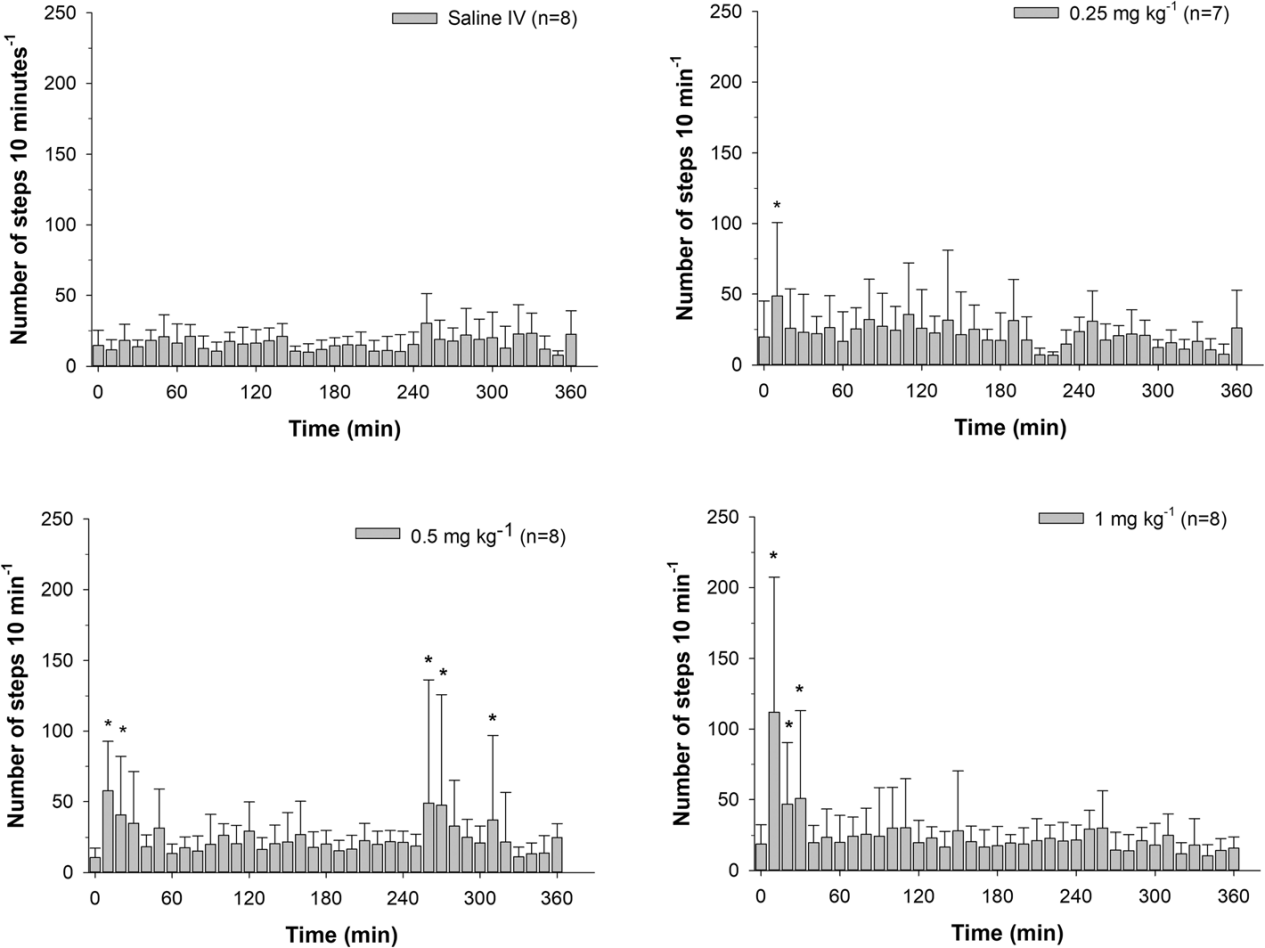
Average ± SDnumber of Steps taken (over a 10-minute period of time) with respect to time following a single IV administration of 0.25, 0.5 and 1.0 mg/kg meperidine HCl to eight horses. *Significant differences relative to time 0 (*p* < 0.05)

The number of steps were not significantly different, relative to baseline, at any time in the saline group. A significant increase in the number of steps was noted during the first 10 minutes in the 0.25 mg/kg dose group, at 10, 20, 260, 270 and 310 minutes following administration of 0.5 mg/kg and at 10, 20 and 30 minutes in the 1 mg/kg dose group (Fig. 4).

Heart rates prior to and following drug administration for all groups are listed in Table 5. Heart rate was significantly (*p* < .05) increased relative to baseline at 2 minutes following administration of 0.5 mg/kg meperidine, and from 2 to 10 minutes and again at 15 minutes post drug administration in the 1 mg/kg dose group. There were no significant differences in respiratory rate, relative to baseline, in any of the dose groups.

**Table 5:** Heart rate (mean ± SD), following a single IV administration of meperidine HCl (0.25, 0.5 and 1.0 mg/kg) to adult horses

Time (h)	Saline	0.25 mg/kg	0.5 mg/kg	1.0 mg/kg
0	33.5 ± 2.8	34.0 ± 2.8	34.0 ± 5.1	34.4 ± 2.3
0.03	33.9 ± 2.4	39.0 ± 8.1	54.3 ± 17.9^*^	90.3 ± 36.4^*^
0.08	34.9 ± 2.5	34.7 ± 3.9	37.6 ± 5.2	50.1 ± 9.3^*^
0.13	35.1 ± 3.0	36.4 ± 4.2	38.5 ± 9.5	50.6 ± 14.0^*^
0.17	35.4 ± 3.2	35.7 ± 3.1	37.1 ± 6.5	44.1 ± 5.2^*^
0.20	34.6 ± 3.1	34.3 ± 1.9	39.3 ± 9.9	40.1 ± 3.4
0.25	35.4 ± 2.8	35.3 ± 4.5	35.9 ± 3.1	44.1 ± 12.9^*^
0.33	34.3 ± 2.1	34.7 ± 3.9	37.9 ± 3.0	41.1 ± 6.1
0.5	34.7 ± 3.2	35.4 ± 2.6	35.8 ± 3.5	37.0 ± 1.4
0.75	33.7 ± 3.0	34.9 ± 2.2	38.6 ± 8.2	36.9 ± 2.5
1	33.6 ± 3.6	33.4 ± 6.4	35.3 ± 2.0	36.8 ± 3.4
1.25	33.7 ± 2.4	36.1 ± 3.3	36.0 ± 3.9	35.9 ± 3.8
1.5	36.9 ± 3.6	35.3 ± 2.7	38.4 ± 6.0	39.6 ± 10.5
2	32.3 ± 4.4	32.7 ± 4.2	34.5 ± 3.7	34.0 ± 4.6
2.5	31.7 ± 4.0	36.0 ± 2.5	35.1 ± 3.1	36.5 ± 4.0
3	36.8 ± 5.4	33.4 ± 5.2	33.1 ± 3.1	32.4 ± 3.9
4	34.8 ± 8.7	34.6 ± 3.5	32.5 ± 2.8	32.0 ± 2.3
5	31.1 ± 4.6	37.9 ± 21.5	35.5 ± 6.6	34.9 ± 5.1
6	31.7 ± 2.7	31.0 ± 3.1	32.9 ± 3.4	31.4 ± 3.2

*, indicates a significant difference (*p* < .05) relative to baseline

Packed cell volume and TP prior to and post meperidine administration are listed in Table 6. There were no significant differences in the saline treated control group. Packed cell volume was significantly decreased from baseline at 10, 15 and 30-minutes following administration of 0.25 mg/kg and significantly increased at 5 and 10 minutes in the 1 mg/kg dose group. Relative to baseline, TP was significantly increased at 45 and 60 minutes in the 0.25 mg/kg dose group and from 30 to 120 minutes following administration of 0.5 mg/kg.

**Table 6:** Packed cell volume (PCV) and total plasma protein (TP), mean ± SD, following a single IV administration of meperidine HCl (0.25, 0.5 and 1.0 mg/kg) to adult horses

Time (h)	Saline (*n* = 8)		0.25 mg/kg (*n* = 7)		0.5 mg/kg (*n* = 8)		1.0 mg/kg (*n* = 8)	
	PCV (%)	TP (g 100 mL^− 1^)	PCV (%)	TP (g 100 mL^− 1^)	PCV (%)	TP (g 100 mL^− 1^)	PCV (%)	TP (g 100 mL^− 1^)
Baseline	35.5 ± 1.4	6.3 ± 0.2	35.5 ± 2.4	6.2 ± 0.4	35.2 ± 2.2	6.2 ± 0.3	36.0 ± 1.9	6.2 ± 0.3
0.08	34.9 ± 1.3	6.4 ± 0.2	34.9 ± 1.9	6.2 ± 0.3	37.0 ± 3.8	6.2 ± 0.3	41.1 ± 6.3^*^	6.2 ± 0.3
0.16	34.8 ± 1.4	6.4 ± 0.2	34.0 ± 1.9^*^	6.2 ± 0.3	35.7 ± 3.6	6.2 ± 0.3	39.4 ± 5.4^*^	6.1 ± 0.3
0.25	35.0 ± 1.9	6.4 ± 0.3	34.0 ± 1.5^*^	6.2 ± 0.3	35.5 ± 2.8	6.2 ± 0.3	38.1 ± 4.6	6.1 ± 0.3
0.5	35.0 ± 2.0	6.4 ± 0.3	33.9 ± 1.1^*^	6.3 ± 0.3	34.6 ± 1.6	6.3 ± 0.3^*^	36.0 ± 3.0	6.1 ± 0.3
0.75	35.4 ± 1.6	6.4 ± 0.3	35.1 ± 2.5	6.4 ± 0.4^*^	36.6 ± 2.8	6.4 ± 0.3^*^	36.3 ± 2.9	6.2 ± 0.4
1	35.3 ± 1.9	6.5 ± 0.3	34.8 ± 1.2	6.4 ± 0.4^*^	35.8 ± 2.0	6.5 ± 0.4^*^	36.7 ± 3.6	6.3 ± 0.4
2	35.5 ± 1.4	6.4 ± 0.2	35.3 ± 1.0	6.3 ± 0.4	35.4 ± 2.3	6.4 ± 0.4^*^	36.2 ± 2.7	6.2 ± 0.5
4	36.2 ± 3.2	6.3 ± 0.3	34.8 ± 1.7	6.3 ± 0.3	35.3 ± 2.5	6.3 ± 0.3	36.1 ± 1.8	6.1 ± 0.4
6	36.1 ± 3.2	6.3 ± 0.2	35.1 ± 1.6	6.2 ± 0.2	35.8 ± 2.5	6.3 ± 0.3	36.0 ± 2.2	6.2 ± 0.4

*, indicates a significant difference (*p* < .05) relative to baseline

Gastrointestinal sounds were significantly decreased compared to baseline in the 1.0 mg/ kg meperidine dose group at 45, 60, 120 and 480-minutes post administration (Table 7). Overall, there was not a noticeable difference in the number of fecal piles, size of the fecal balls within a pile or consistency between dose groups.

**Table 7:** Gastrointestinal scores (mean ± SD), following a single IV administration of meperidine HCl (0.25, 0.5 and 1.0 mg/kg) to adult horses

Time (h)	saline	0.25 mg/kg	0.5 mg/kg	1.0 mg/kg
Baseline	2.4 ± 1.2	2.4 ± 1.4	2.5 ± 1.3	3.0 ± 1.4
0.5	1.9 ± 0.4	2.6 ± 0.5	2.6 ± 1.1	2.5 ± 1.4
0.75	2.4 ± 0.9	2.3 ± 1.0	2.6 ± 0.5	2.0 ± 0.9^*^
1	2.5 ± 1.1	2.6 ± 0.5	2.9 ± 1.4	2.1 ± 0.4^*^
2	2.3 ± 0.9	2.6 ± 1.0	2.5 ± 0.8	2.1 ± 0.8^*^
4	1.9 ± 0.6	2.6 ± 1.0	2.4 ± 0.9	2.5 ± 1.4
6	2.3 ± 0.9	2.1 ± 0.9	2.4 ± 1.3	2.6 ± 0.5
8	---	3.0 ± 1.2	3.1 ± 1.6	1.9 ± 0.6^*^
24	---	2.7 ± 1.3	2.7 ± 1.1	2.8 ± 1.2

---, not assessed; *, indicates a significant difference (*p* < .05) relative to baseline

### Thermal Threshold

Skin and ambient temperature, thermal threshold and the %TE at each time point are listed in Table 8. There were no significant differences in the thermal threshold or the %TE at any time post saline administration or following administration of 0.25 mg/kg meperidine, compared to baseline (Table 8). A significant increase in both the thermal threshold and the %TE was observed at 15, 30 and 45-minutes post drug administration in the 0.5 mg/kg, and at 15 minutes post administration in the 1 mg/kg dose group (Table 8).

**Table 8:** Skin temperature, thermal threshold (TT) and thermal excursion (%TE) and ambient temperature following a single IV administration of saline, 0.25, 0.5 and 1.0 mg/kg meperidine HCl to 8 horses. Values are expressed as mean ± SD

Dose Group					Time (h)						
	0	0.25	0.5	0.75	1	1.5	2	3	4	6	
Skin Temp (°C)											
Saline	24.6 ± 3.1	25.2 ± 3.1	26.3 ± 3.4^*^	26.7 ± 3.2^*^	27.3 ± 3.3^*^	28.2 ± 3.1^*^	29.3 ± 2.9^*^	30.5 ± 1.9^*^	32.0 ± 2.0^*^	32.0 ± 2.0^*^	
0.25 mg/kg	24.8 ± 1.7	24.8 ± 2.3	25.9 ± 2.5	26.2 ± 2.8	28.1 ± 3.3^*^	28.5 ± 3.6^*^	29.2 ± 4.0^*^	28.8 ± 4.2^*^	29.7 ± 4.0^*^	30.6 ± 4.6^*^	
0.5 mg/kg	25.4 ± 3.2	25.4 ± 3.7	27.4 ± 3.8^*^	28.1 ± 4.0^*^	29.7 ± 2.4^*^	30.4 ± 1.6^*^	31.3 ± 1.0^*^	31.6 ± 0.9^*^	32.1 ± 1.0^*^	32.1 ± 1.4^*^	
1 mg/kg	25.4 ± 2.3	25.9 ± 2.8	26.5 ± 3.3	27.5 ± 2.8^*^	28.7 ± 1.7^*^	31.3 ± 3.8^*^	30.8 ± 1.0^*^	30.3 ± 1.7^*^	30.7 ± 2.1^*^	32.1 ± 0.8^*^	
TT (°C)											
Saline	48.5 ± 4.9	50.0 ± 4.2	48.6 ± 6.9	46.7 ± 6.5	50.0 ± 7.8	47.6 ± 3.7	49.9 ± 3.7	50.0 ± 6.3	49.4 ± 4.8	50.6 ± 4.7	
0.25 mg/kg	49.6 ± 3.8	50.3 ± 3.2	46.9 ± 6.9	51.4 ± 4.3	51.0 ± 4.8	51.7 ± 4.3	49.5 ± 5.4	50.7 ± 5.0	51.4 ± 5.9	52.8 ± 3.3	
0.5 mg/kg	47.7 ± 6.0	51.7 ± 3.6^*^	50.5 ± 4.5^*^	50.8 ± 5.5^*^	49.9 ± 4.0	47.4 ± 5.3	47.4 ± 4.3	48.7 ± 5.9	51.5 ± 4.0^*^	49.7 ± 3.7	
1 mg/kg	49.0 ± 3.4	53.5 ± 2.6^*^	51.3 ± 3.6	48.9 ± 5.6	50.4 ± 3.1	50.6 ± 5.5	47.8 ± 4.9	47.9 ± 3.1	49.3 ± 4.3	48.9 ± 6.4	
% TE											
Saline	79.4 ± 15.2	83.4 ± 13.2	78.6 ± 21.8	70.5 ± 24.5	81.9 ± 28.3	71.6 ± 14.1	79.8 ± 14.6	78.9 ± 26.7	75.6 ± 21.4	80.2 ± 21.2	
0.25 mg/kg	82.1 ± 12.2	84.3 ± 10.8	70.6 ± 26.0	87.4 ± 15.1	84.2 ± 18.7	86.2 ± 17.6	76.4 ± 23.1	82.9 ± 18.5	84.9 ± 24.3	90.1 ± 14.3	
0.5 mg/kg	75.4 ± 20.9	88.6 ± 12.8^*^	84.2 ± 15.7^*^	84.9 ± 20.7^*^	79.7 ± 16.0	69.3 ± 21.4	67.7 ± 18.3	73.1 ± 25.3	84.6 ± 17.9	76.8 ± 16.5	
1 mg/kg	79.7 ± 11.3	94.9 ± 8.9^*^	87.3 ± 12.4	78.4 ± 19.0	82.4 ± 12.1	81.6 ± 23.0	70.2 ± 20.4	71.1 ± 12.5	76.6 ± 18.0	73.3 ± 27.2	
Ambient Temp (°C)											
Saline	19.4 ± 1.2	20.0 ± 1.0^*^	20.1 ± 1.0^*^	20.3 ± 1.1^*^	20.4 ± 1.0^*^	20.8 ± 1.0^*^	21.1 ± 1.1^*^	22.2 ± 1.2^*^	23.4 ± 1.4^*^	25.8 ± 1.4^*^	
0.25 mg/kg	20.9 ± 1.9	20.8 ± 1.8	20.9 ± 1.9	21.1 ± 1.9	21.1 ± 1.9	21.4 ± 2.0	21.8 ± 2.1^*^	22.6 ± 2.2^*^	23.6 ± 2.0^*^	25.0 ± 1.3^*^	
0.5 mg/kg	19.9 ± 2.1	20.2 ± 1.9	20.4 ± 2.0	20.7 ± 2.0	20.9 ± 2.0^*^	21.4 ± 1.9^*^	21.9 ± 1.9^*^	22.9 ± 2.0^*^	23.8 ± 2.0^*^	24.7 ± 1.5^*^	
1 mg/kg	20.5 ± 1.4	20.6 ± 1.2	20.8 ± 1.2	20.9 ± 1.2^*^	21.1 ± 1.2^*^	21.6 ± 1.2^*^	22.1 ± 1.1^*^	23.1 ± 1.2^*^	24.1 ± 1.1^*^	26.1 ± 1.4^*^	

*, indicates a significant difference (*p* < .05) relative to baseline

## Discussion

The goal of the current study was to describe the pharmacokinetics of meperidine following IV administration as well as the effects of the drug on select pharmacodynamic effects, including thermal nociception. Non-compartmental modeling was utilized for calculation of pharmacokinetic parameters due to apparent dose dependent pharmacokinetics. Specifically, an increase in the V_ss_ and total systemic clearance was observed with increasing dose. The V_ss_ is dependent on drug factors (lipophilicity, plasma protein binding) and as such, changes may be observed as a result of differences in physiologic variables (age, body composition). In the current study, the same animals were used in each dose group and the same drug formulation (no change in physiochemical properties) was administered, therefore changes in V_ss_ were unexpected. Although, it is not possible to definitely determine the reason for the increase in V_ss_, the most likely explanation is changes related to plasma protein and/or tissue binding. While no studies were found specifically describing changes in V_ss_ following administration of escalating doses of opioids, there are reports of increases in V_ss_ for other drugs, which have been attributed to changes in protein and/or tissue binding.

Non-linear changes in clearance are most often reported as decreasing clearance with increasing doses, oftentimes a result of saturation of a process that contributes to elimination. Conversely, in the current study, clearance increased with increasing doses. While relatively uncommon, this finding is similar to those in reports of the pharmacokinetics of other opioids, specifically hydromorphone and morphine, following administration to horses [18, 19]. In these studies, the investigators theorized that the increase in clearance with changes in dose was a result of increasing hepatic blood flow, and thus an increase in hepatic clearance of these high extraction ratio drugs [18, 19]. Similar to morphine and hydromorphone, meperidine is classified as a high-extraction ratio drug [20]. Although cardiac output was not assessed in the current study it is possible that the drug induced increase in heart rate may have led to an increase in cardiac output. A resultant increase in hepatic blood flow would then be the most likely explanation for the increased clearance noted with increasing meperidine doses.

As described in other species, in the current study, normeperidine was detected in plasma samples collected from all horses with metabolite concentrations increasing in a dose dependent fashion. In other species, higher normeperidine concentrations have been associated with an increased incidence of CNS excitation [16, 17, 21, 22]. In the current study, while notable behavioral effects were observed following administration of all doses of meperidine and effects varied greatly between individual horses, evidence of CNS excitation (circling, trotting around the stall) was most pronounced following administration of higher meperidine doses. Closer visual analysis of the results presented here suggests that transient increases in quantitative measures associated with CNS stimulation (e.g. step count, HR and PCV) occur at plasma meperidine concentrations above approximately 500 ng/mL regardless of the meperidine dose administered. Similarly, the approximate corresponding plasma normeperidine concentration as 4–5 ng/ mL. Although further study would be necessary, including administration of normeperidine, it is possible that the CNS excitation observed following high dose meperidine administration was caused by higher concentrations of normeperidine. While a significant increase in the number of steps taken was noted at 260, 270 and 310 minutes post administration at the 0.5 mg/kg meperidine dose (Fig. 3), the statistical significance likely resulted from a heightened reaction coinciding with external stimulation (e.g. noise/activity outside the individual horse’s stall) and were therefore deemed to be a result of abrupt changes in environmental conditions, as opposed to drug-induced activity.

At the intermediate (0.5 mg/kg) and high dose (1.0 mg/kg), most horses demonstrated a cutaneous reaction similar to what has been reported following both meperidine and morphine administration in other species [3, 23]. This type of reaction has been attributed to drug-induced release of histamine by what is reportedly a mu-receptor independent mechanism [24, 25]. In the current study, the cutaneous reaction started within 30–60 seconds of administration and included sweating and urticaria that quickly spread over the entire body and caused what appeared to be extreme itchiness. Similarly, in humans, cutaneous responses were observed as early as one-minute post IV administration of meperidine, corresponding with peak concentrations of histamine [26].

Nociceptive threshold testing, including assessing the effects of drugs on thermal and/or mechanical stimuli, is a commonly used experimental approach to assess the efficacy of analgesic agents. With advances in technology, thermal threshold testing is increasingly reported in equine studies. In the current study, a previously well-described model of thermal nociception in horses [27, 28] was utilized to assess the effects of different doses of meperidine on thermal thresholds and the %TE. This same model was used in a previous study describing the antinociceptive effects of another opioid, hydromorphone in horses [19]. Reed and colleagues [19] described a pronounced and extended (12 hours) duration of effect on thermal nociception following IV administration of 0.04 and 0.08 mg/kg hydromorphone to horses [19]. Albeit for a shorter period of time than in the study conducted by Reed and colleagues [19], similar dose dependent increases in thermal nociception were observed following administration of morphine, butorphanol and levomethadone to horses [29]. These findings and those reported by Reed and colleagues [19] are in contrast to the current study, whereby increases in the thermal threshold were short in duration and observed only in the two higher dose groups. It should be noted, however, that the shorter duration of action is consistent with reports of a shorter duration of analgesic effect for meperidine, compared to many other opioids, in other species [4]. Excepting for the brief report by Steffey and Pascoe [7] there are no published studies describing the effects of meperidine on nociception in horses. However, using a model similar to that described for the currently reported study similar observations on thermal nociception have been reported following administration of a constant rate infusion of butorphanol [30] and IV administration of methadone to horses.

Although, 1 mg/kg has been previously reported to be a clinically acceptable dose, there are only minimal data to support this [31]. While notably, thermal nociception may not be representative of clinical pain and the behavioral responses in sick or injured horses may be different, in the current study, a longer duration of effect was observed at 0.5 mg/kg compared to 1 mg/kg. While the reason for this is not immediately apparent, it may be related to CNS stimulation observed when horses are administered a dose of 1 mg/kg. This stimulation may counteract the anti-nociceptive effect and/or confound data interpretation. Regardless the notable adverse effects, specifically signs of CNS excitation and cutaneous responses do not support the use of IV meperidine at 1 mg/kg, at least not without individual horse consideration and/or concurrent administration of a drug with sedative or tranquilizer properties.

## Conclusions

Results of the current study do not support routine clinical use of IV meperidine at a dose of 1 mg/kg (resulting in an average ± SD measured C(0) of 987.7 ± 224 ng/mL) to horses, and although administration of 0.5 mg/kg (similarly, an average ± SD C(0) of 691.9 ± 123 ng/mL) may provide short-term analgesia, the associated inconsistent and/or short term adverse effects suggest that its use as a sole agent at this dose, at best, must be cautiously considered. While no notable adverse effects were observed following intravenous administration of 0.25 mg/kg meperidine, there was not a significant effect on thermal nociception.

## Methods

### Horses

Eight healthy university-owned thoroughbreds, four mares and four geldings (aged 3–8) with an average ± SD weight of 533.7 ± 29.2 kg were used for this study. The number of horses selected for this study was based on pharmacodynamic endpoints, specifically thermal threshold. A power analysis assuming a mean baseline thermal excursion of 71, a mean thermal excursion of 95 in each treatment group immediately after treatment, and a mean thermal excursion of 85 in each treatment group 1-hour post-treatment, using data from a related study [32] was conducted. For paired t-tests, a standard deviation of 10 for the difference between baseline and treatment was assumed. Based on these assumptions, a sample size of 4 horses is sufficient to detect the difference between baseline and initial treatment, and a sample size of 8 horses should be sufficient to detect the difference between baseline and 1-hour post-treatment.

Horses were not administered any medications for a minimum of two weeks before the study. A complete blood count, serum biochemistry, and physical exam were performed prior to the start of the study to assess the health status of the horses. Horses were housed in 12 × 12 stalls in a temperature-controlled barn, starting 2 days before the start of the study and for a minimum of 48 hours following drug administration. Breezeway doors remained closed throughout the duration of the study and personnel access was limited to decrease the influence of external factors on horse behavior. The Institutional Animal Care and Use Committee of the University of California, Davis approved this study. Following completion of the study, horses returned to the research herd at the University of California, Davis.

### Instrumentation and drug administration

The study was conducted in a balanced 4-way crossover design with a minimum two-week washout between treatments. The order of treatment was randomized for individual horses using a randomized number generator. A single IV dose of either 0.25, 0.5 or 1 mg/kg meperidine HCL (Demerol, Pfizer, New York, NY, USA) or 5 mL of saline was administered. A 14-gauge catheter was placed in each jugular vein, using sterile technique, prior to drug administration. One catheter was used for drug administration, while the contralateral catheter was used for sample collection.

### Sample Collection

Blood, for determination of meperidine and normeperidine concentrations was collected immediately prior to drug administration (time 0) and at times 5, 10, 15, 30, 45 minutes, and 1, 1.5, 2, 2.5, 3, 4, 5, 6, 8, 12, 18, 24, 36, 48, and 96 hours following drug administration as described previously [18]. Sampling catheters were removed after collection of the 18-hour sample with the remaining samples collected via direct venipuncture. Blood samples were centrifuged at 3000 x g at 4 °C for 10 minutes, plasma was immediately transferred to cryovials (Phoenix Research Products, Chandler, NC, USA) and samples stored until analysis at -20 °C.

Prior to centrifugation, an aliquot (500 ul) was taken from the EDTA tubes for determination of packed cell volume (PCV) and total protein (TP) at 0 (immediately before administration), 5, 10, 15, 30, 45 minutes, 1, 2, 4, and 6 hours post-drug administration. Packed cell volume was measured via microhematocrit determination, while TP was measured via refractometer. Each sample was measured in duplicate with the average of the two readings recorded for each time point.

Urine was collected for determination of meperidine and normeperidine concentrations at 4, 24, 48, 72, and 96 hours post drug administration by free catch then stored at -20 °C until analysis. The exact time of sample collection was recorded.

### Plasma Concentration Determination

Meperidine and normeperidine (Cerilliant, Round Rock, TX, USA) were combined into one working solution by dilution of the stock solutions with methanol to concentrations of 10, 100, 1000, 10,000 and 100,000 ng/mL. Working standard solutions were diluted with drug free equine plasma for preparation of plasma calibrators with the standard curve concentrations ranging from 0.1 to 2000 ng/mL. Calibration curves and negative control samples were prepared fresh for each assay. Quality control samples (equine drug free plasma fortified with analyte at three concentrations within the standard curve, high, medium and low (3X LOQ of the assay)) were included with each sample set.

Prior to analysis, 500 µL of plasma was diluted with 500 µL of ACN:1M acetic acid (9:1, v:v) containing 0.01 ng/uL of the internal standards d4-meperidine and d4-normeperidine (Cerilliant, Round Rock, TX, USA), to precipitate proteins. The samples were vortexed for 2 minutes to mix, refrigerated for 20 minutes, vortexed for an additional 1 minute, centrifuged at 4300 rpm/3830 g for 10 minutes at 4 ºC and 20 µL injected into the liquid chromatography tandem mass spectrometry (LC-MS/MS) system.

The concentrations of meperidine and normeperidine were measured in plasma by LC-MS/MS using positive heated electrospray ionization (HESI(+)). Quantitative analysis of plasma was performed on a TSQ Vantage triple quadrupole mass spectrometer (Thermo Scientific) having an LC-10ADvp liquid chromatography system (Shimadzu, Kyoto, Japan). The spray voltage was 3500V, the vaporizer temperature was 362 ºC, and the sheath and auxiliary gas were 45 and 25 respectively (arbitrary units). Product masses and collision energies were optimized by infusing the standards into the mass spectrometer. Chromatography employed an ACE 3 C18 10 cm x 2.1 mm column (Mac-Mod Analytical, Chadds Ford, PA, USA) and a linear gradient of acetonitrile (ACN) in water with 0.2% formic acid, at a flow rate of 0.40 ml/min. The initial ACN concentration was held at 10% for 0.42 minutes, ramped to 50% over 5.83 minutes, ramped to 95% over 0.17 minute, before re-equilibrating for 3.67 minutes.

Detection and quantification was conducted using selective reaction monitoring (SRM) of initial precursor ion for meperidine (mass to charge ratio *(m/z)* 248.1), normeperidine (*(m/z)* 234.1), and the internal standards d4-meperidine (*(m/z)* 252.1), d4-normeperidine (*(m/z)* 238.1). The response for the product ions for meperidine (*m/z* 91, 103, 220), normeperidine (*m/z* 160), and the internal standards d4-meperidine (*m/z* 224), d4-normeperidine (*m/z* 164) were plotted and peaks at the proper retention time integrated using Quanbrowser software (Thermo Scientific). Quanbrowser software was used to generate calibration curves and quantitate analytes in all samples by linear regression analysis. A weighting factor of 1/X was used for all calibration curves.

The technique was optimized to provide a limit of quantitation (LOQ) of 0.1 ng/ mL and a limit of detection (LOD) of approximately 0.05 ng/mL for both meperidine and normeperidine.

### Urine Concentration Determination

The same working solutions were used for urine as described for plasma above. Briefly, urine calibrators were prepared by dilution of the working standard solutions with drug free equine urine to concentrations ranging from 0.25 to 5000 ng/mL. Calibration curves and negative control samples were prepared fresh for each quantitative assay. In addition, quality control samples (equine drug-free urine fortified with analyte at three concentrations within the standard curve) were included with each sample set as an additional check of accuracy.

Prior to analysis, 0.5 mL of urine was diluted with 200 µL of water containing 0.01 ng/uL of d4-meperidine and d4-normeperidine and 0.2 mL of β-glucuronidase enzyme, (Sigma Aldrich, St. Louis, MO, USA) at 10,000 Units/mL in pH 5, 1.6 M acetate buffer and the samples vortexed briefly to mix. The pH of samples was adjusted to 5 ± 0.5 with 2 N NaOH or 2 N HCl, as necessary, and heated in a sonicating water bath at 65 °C for 2 hours with 99 minutes of sonication. After cooling to room temperature, 2 mL of 0.6 M, pH 6.5 phosphate buffer was added and the samples were subjected to solid phase extraction using Cerex polycrom Clin II 3 cc 35 mg columns (Cera, Inc. Baldwin Park, CA, USA). Samples were loaded onto the columns, washed with 3 mL of water followed by 2 mL of 1 M acetic acid before rinsing with 3 methanol and eluting with 2.5 mL of 78:20:2 (v:v:v) methylene chloride:isopropanol:ammonium hydroxide. Samples were dried under nitrogen, dissolved in 200 µL of 5% ACN in water with 0.2% formic acid and 20 µL injected into the LC/MS/MS system.

Detection and quantification were the same as described above for plasma except the meperidine product ions used for quantitation were (*m/z* 91.1, 103.1). The technique was optimized to provide an LOQ of 0.25 ng/mL and a LOD of approximately 0.1 ng/ mL for both meperidine and normeperidine.

### Pharmacokinetic Calculations

Previous studies have demonstrated non-linearity with respect to clearance of opioids in horses [18, 19]. Therefore, non-compartmental analysis was performed on plasma meperidine concentrations using a commercially available software (Phoenix WinNonlin Version 8.1, Certara, Princeton, NJ, USA) to assess whether similar behavior was seen with respect to meperidine clearance. Lambda *z* (λ_*z*_) was used to calculate the terminal half-life (HL λ_z_) using the Eq. 0.693/ λ. The area under curve (AUC) from time 0 to infinity (AUC_0→∞_) was obtained by using the linear up log down trapezoidal rule. Clearance (Cl) and the apparent volume of distribution at steady state (V_ss_) were determined using the following formulas:

$$ CI=Dose/AOC_{0\rightarrow\infty} $$

$$ {V}_{ss}={MRT}_{inf}x\; Cl $$

where MRT is the mean residence time.

Non-compartmental analysis, as described above was also used for determination of pharmacokinetic parameters for normeperidine.

### Physiologic Responses

Prior to drug administration, horses were equipped with two Step Monitors (SAM3, Seattle, WA, USA) programmed to count the number of steps taken each minute [18]. Holter monitors (Forrest medical, East Syracuse, NY, USA) were also used on each horse to assess any potential effects of the drugs on heart rate [18].

Gastrointestinal sounds, were assessed as described previously [18], prior to and at 30 and 45 minutes, as well as 1, 2, 4, 6, 8, and 24 hours post-drug administration. The number of fecal piles, number of fecal balls within a pile and fecal consistency were recorded prior to drug administration (included 2-4-hour period prior), at 2, 4, 6, 8, 12 and 24 hours. All fecal piles were removed from the stall after characteristics were recorded at each time point. Pile size was considered small if less than 15 fecal balls, average if 15–30 and large if greater than 30. Consistency was recorded as normal, wet or dry.

Additional notable physiological or behavioral observations were noted throughout the sampling period.

### Thermal Threshold Determination

Thermal threshold testing was conducted using a commercially available wireless device WTT2; TopCat Metrology, UK) as described previously [32]. Briefly, an area on the outside of the metacarpus was shaved at least one day prior to applying the temperature probe. The temperature probe was placed in direct contact with the skin and the nylon strap tightened around the leg. To ensure proper and consistent contact with the leg, 10 cc of air was injected into an air bladder attached to a psi gauge. The probe was placed on top of the air bladder against the leg. The Velcro strap was then tightened until the gauge read 80psi. The air bladder was subsequently removed so that the thermal probe laid directly against the skin. Skin temperature at the location of the thermal element was noted prior to each reading as was the ambient temperature. The change in temperature was controlled by an operator via an infrared remote outside of the stall. When activated by a button, the thermal element heated at a rate of 1.1 °C per second until the horse responded to the stimulus by stomping, lifting, pawing with or touching their nose to the right front leg. The temperature at which the horse responded to the stimulus (stomping, lifting, pawing with or touching their nose to the right front leg) was recorded as the threshold temperature for the time point. A “trip” temperature (temperature the unit would automatically shut off and terminate heating) of 55 °C is automatically set on the machine to avoid tissue burns. Baseline response measurements were taken every other day for one week leading up to drug administration to acclimate the horse to the machine and characterize the individual horse’s response. Baselines were also taken in triplicate the morning of drug administration with a 5-minute interval between each reading. Thermal readings were obtained 15, 30, 45, 60, 90, 120, 180, 240 and 360 minutes after administration of meperidine or saline.

### Statistics

For comparability of treatments, thermal nociceptive thresholds were standardized to thermal exclusion (%TE) as described previously [32], using the formula:

$$ \text{\%TE}=100\times{\left[(\text{T}_{\text{T}}-\text{T}_{0})/\left(\text{T}_{\text{c}}-\text{T}_{0}\right)\right]} $$

Where T_T_ represents the thermal threshold, T_0_ the skin temperature and T_C_ the thermal nociceptive cut-off temperature.

Statistical analyses using commercially available software (Stata/IC 15.1, StataCorp LP, TX, USA) were used to determine significant differences in pharmacokinetic and pharmacodynamic parameters. For pharmacokinetic analysis, differences in parameters between dose groups were assessed and for pharmacodynamic data, differences between baseline and each time point and between dose groups were evaluated. Data were analyzed using a mixed effects analysis of variance, with the horse as the random effect and time and dose as the fixed effects. Post-hoc comparisons were performed with a Bonferroni multiple comparison adjustment to preserve a nominal significance level of 0.05.

## Data Availability

The datasets used and/or analyzed during the current study are available from the corresponding author on reasonable request.

## Abbreviations

PCV: Packed Cell Volume
TP: Total Protein
LC-MS/MS: Liquid Chromatography Tandem Mass Spectrometry
ACN: Acetonitrile
SRM: Selective Reaction Monitoring
LOQ: Limit of Quantitation
LOD: Limit of Detection
AUC: Area Under Curve
Cl: Clearance
V_ss_: Volume of distribution at steady state
%TE: Thermal Exclusion
T_T_: Thermal Threshold
T_0_: Skin Temperature
T_C_: Thermal nociceptive cut-off temperature
